# Improved Cartilage Protection with Low Molecular Weight Hyaluronic Acid Hydrogel

**DOI:** 10.3390/bioengineering10091013

**Published:** 2023-08-27

**Authors:** Riley B. Brackin, Gail E. McColgan, Saitheja A. Pucha, Michael A. Kowalski, Hicham Drissi, Thanh N. Doan, Jay M. Patel

**Affiliations:** 1 Atlanta VA Medical Center, Decatur, GA 30033, USA; 2 Department of Orthopaedics, Emory University School of Medicine, Atlanta, GA 30329, USA

**Keywords:** cartilage, hyaluronic acid, biomaterials, joint preservation

## Abstract

Traumatic joint injuries are common, leading to progressive tissue degeneration and the development of osteoarthritis. The post-traumatic joint experiences a pro-inflammatory milieu, initiating a subtle but deteriorative process in cartilage tissue. To prevent or even reverse this process, our group previously developed a tissue-penetrating methacrylated hyaluronic acid (MeHA) hydrogel system, crosslinked within cartilage to restore and/or protect the tissue. In the current study, we further optimized this approach by investigating the impact of biomaterial molecular weight (MW; 20, 75, 100 kDa) on its integration within and reinforcement of cartilage, as well as its ability to protect tissue degradation in a catabolic state. Indeed, the low MW MeHA integrated and reinforced cartilage tissue better than the high MW counterparts. Furthermore, in a 2 week IL-1β explant culture model, the 20 kDa MeHA demonstrated the most protection from biphasic mechanical loss, best retention of proteoglycans (Safranin O staining), and least aggrecan breakdown (NITEGE). Thus, the lower MW MeHA gels integrated better into the tissue and provided the greatest protection of the cartilage matrix. Future work will test this formulation in a preclinical model, with the goal of translating this therapeutic approach for cartilage preservation.

## 1. Introduction

Traumatic joint injuries are exceedingly common; for the knee alone, there are more than 200 k anterior cruciate ligament (ACL) injuries [1] annually in the United States, with similar rates of injury to the meniscus [2]. These injuries alter the biomechanical and biological status of the joint, placing a greater burden on the articular cartilage [3,4] and leaving it highly susceptible to breakdown. A gradual cycle of wear and tear ensues layer by layer, eventually leading to post-traumatic osteoarthritis (PTOA) [5]. PTOA affects tens of millions of patients, causes pain, disability, and decreased quality of life, and frequently necessitates total joint replacement. Even successful tissue reconstructions (e.g., ACL reconstruction and meniscal repair) do not impact disease evolution; more than 50% of ACL reconstructions (ACLR) still lead to PTOA [6]. Overall, OA is the leading cause of work disability and contributes an estimated $303.5 billion in combined medical care costs and losses annually [7]. Thus, early preventative measures are required to delay this progression, specifically as they relate to preserving the articular cartilage. 

Following joint injuries and the subsequent reparative surgeries, a sudden and strong biological cascade is observed in joints. The release of inflammatory cytokines (e.g., TNF-α, IL-1β, IL-6, and IL-10; [8,9]) into the synovial fluid typically persists for weeks to months [10,11,12,13]. Furthermore, the milieu in the synovial fluid spreads to tissues across the joint, including articular cartilage [14,15], leading chondrocytes within the tissue to produce matrix metalloproteinases (MMPs) and a disintegrin and metalloproteinase with thrombospondin motifs (ADAMTS), among other degradative enzymes. This activity initiates a loss of matrix components, mostly collagen and proteoglycans. These subtle but irreversible tissue changes are unrecognized arthroscopically [9] or radiographically; early tissue loss is histologically characterized by loss of proteoglycan staining and changes in cellularity, and functionally characterized by deficits in biphasic biomechanical properties (reduced modulus and increased permeability [16,17]). These shifts leave the cartilage susceptible to future biophysical or catabolic events [18], often initiating a degenerative feedback loop that results in OA progression. 

To combat joint inflammation and degeneration, physicians have used a variety of bioactive factors (e.g., NSAIDS and hyaluronic acid [HA]) and biologics (platelet-rich-plasma [PRP] and mesenchymal stromal cells [MSCs]) [19]. However, their residence time within the synovial fluid may only be days [20,21,22], and in a catabolic state, hours. For this reason, researchers have developed therapies that more effectively penetrate and impact tissue properties. For example, cationic nanoparticles and peptides can effectively deliver anti-inflammatory molecules or reactive oxidative species (ROS) scavengers within the negatively charged cartilage tissue [23,24,25], reducing cartilage degradation in inflammatory and post-traumatic OA models. Beyond drug delivery, a few groups have aimed to reinforce or replenish the weakened cartilage matrix; approaches have included chemical crosslinking [26,27], interpenetrating synthetic polymer networks [28,29], and biomimetic proteoglycans [30,31]. Hydrogels, in particular, are of great interest since they are tunable systems utilized for both cartilage preservation and tissue engineering [32,33]. In our previous study [34], we developed a modified hyaluronic acid (HA) system that simultaneously reinforced cartilage and reduced catabolic processes. These advancements in matrix-penetrating therapies hold promise for mitigating the progression of OA and warrant further investigation in the quest for effective treatment strategies.

**Figure 1:**
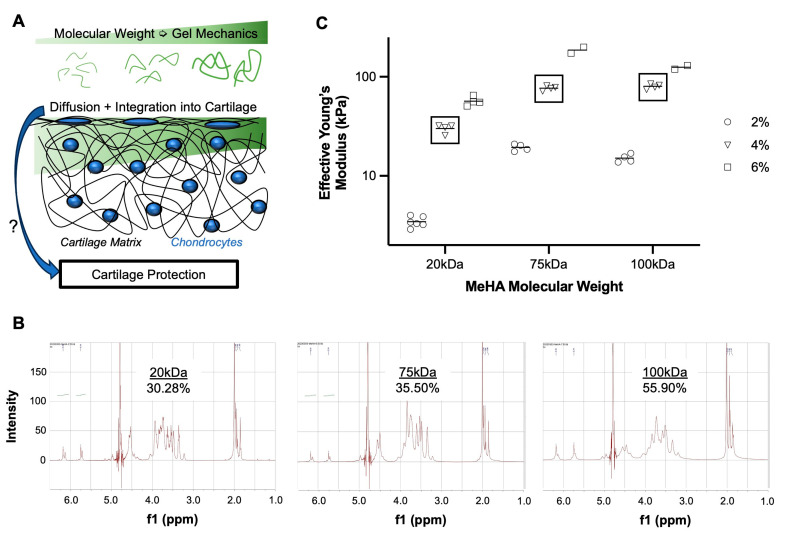
Tissue-infiltrating methacrylated hyaluronic acid hydrogel system. (**A**) Gel mechanics and diffusion of biomaterial into the cartilage matrix are molecular-weight dependent, and their influence on cartilage protection is unclear. (**B**) NMR spectroscopy of MeHA polymers (20, 75, 100 kDa) with calculated degree of methacrylation. (**C**) Effective Young’s Modulus of MeHA gels of different MW (20, 75, 100 kDa) and concentrations (2, 4, 6% *w*/*v*). Square boxes highlight 4% *w/v* formulations utilized for the remainder of studies. Each point represents its own fabricated gel, with ~25 indentations per gel. *n* = 2–5 gels per group.

The diffusion, integration, and efficacy of these cartilage-penetrating approaches are reliant upon several factors [35,36], including, but not limited to, time of application, concentration, charge, cartilage health, and molecular weight (MW). The MW of HA in particular is of great interest, given that current visco-supplementation techniques utilize high MW (1.5–6 MDa), similar to that observed in healthy synovial fluid. However, HA of this size is too large to penetrate within the dense cartilage matrix; for this reason, we previously utilized a 75 kDa methacrylated HA (MeHA) that could diffuse into cartilage and undergo photo-crosslinking to create a tissue-integrative treatment. However, one gap of exploration that remains is the impact of MeHA MW on integration within the tissue and its protective abilities. Certainly, lower MW molecules are expected to diffuse to a greater extent, but higher MW MeHA, when gelled, exhibits greater mechanical properties [37]. Thus, an interplay between MeHA diffusion and reinforcing abilities may exist (Figure 1A) and affect the therapeutic potential of this approach. Thus, the objective of this study was to evaluate three different MW MeHA formulations (20, 75, 100 kDa) with regard to their (1) diffusion and integration within cartilage, (2) time-zero reinforcing capabilities, and (3) anti-catabolic effects in a degenerative explant model.

## 2. Materials and Methods

### 2.1. Experimental Design

To test our hypotheses, we first synthesized methacrylated hyaluronic acid (MeHA) of three different molecular weights (20, 75, and 100 kDa), verifying the degree of methacrylation, and characterizing the mechanical properties of the gels alone. Next, we tested the diffusive integration of the MeHA formulations in bovine cartilage explants, via cross-sectional staining and quantification. Third, we evaluated the time-zero biomechanical augmentation that the hydrogels provide to cartilage, both in terms of restoration (application of MeHA after collagenase-mediated degeneration) and protection (MeHA application prior to collagenase degeneration). Finally, in a degenerative explant model (IL-1β-induced catabolism), we investigated the ability of the three MeHA formulations to prevent biomechanical and biochemical loss.

### 2.2. Hyaluronic Acid Methacrylation and Characterization

MeHA was synthesized from sodium hyaluronate (20 kDa and 75 kDa, LifeCore Biomedical) or obtained from a commercial vendor (100 kDa, PhotoHA, Advanced Biomatrix). For in-house synthesis, sodium hyaluronate was dissolved in deionized water (10 mg/mL, 1% *w*/*v*), to which a 10-fold molar excess of methacrylic anhydride was added. The reaction was maintained at a pH of 8.0–9.0 for 6 h on ice. The reaction was terminated using vigorous overnight stirring at room temperature. The solution was dialyzed (MWCO: 6500 Da) against deionized H_2_O for 7 days, frozen at −20 °C for 24 h, and lyophilized (−50 °C, 0.05 mbar) for 4 days to produce dry MeHA. Proton (^1^H) nuclear magnetic resonance (NMR) spectroscopy was utilized to quantify the degree of methacrylation; MeHA polymer (7–10 mg) was dissolved in deuterium oxide (D_2_O) in thin-walled NMR tubes and imaged at the Emory NMR Core. In Mnova software, we averaged the integration of the peaks at δ 6.2 ppm and δ 5.8 ppm and divided it by the integration of δ 3.0–4.2 ppm (multiplied by 10 protons) to obtain the polymer percent methacrylation [38]. 

Finally, we characterized the mechanical properties of the hydrogel formulations alone. Hydrogel precursors of all three MW were made at 2%, 4%, and 6% *w/v* in phosphate-buffered saline (PBS) with 0.05% Lithium phenyl-2,4,6-trimethylbenzoylphosphinate (LAP) crosslinker. Cylindrical molds (6 mm diameter × 1 mm thickness) were used to fabricate gels, which were crosslinked for 10 min with blue light (400–500 nm, 25 mW/cm^2^, Omnicure S2000). Gel mechanics were characterized using a nano-indentation system (Optics 11 Chiaro). A 10- µm radius probe (~0.5 N/m) was used to test gels to an indentation of 1 μm. The resulting load-deformation curves were fitted with a Hertzian biphasic indentation equation to obtain an effective Young’s modulus. Multiple (2–6) gels of each MW-% combination were tested, with ~25 indentations averaged per gel to obtain gel stiffness.

### 2.3. Explant Dissection and Processing 

Cartilage explants were extracted from the trochlear groove of juvenile bovine stifles (1–3 weeks old, Research 87). Using a cylindrical biopsy punch, full-thickness chondral samples were obtained. After harvest, the top 3 mm of cartilage was separated from the underlying calcified cartilage layers to obtain relatively consistent cartilage samples. Due to the varying properties (thickness, mechanics) of cartilage medially and laterally, we randomized and accounted for the control and experimental groups with respect to the locations on the groove. We also utilized explants from at least three donors per experiment. Explants were either immediately frozen for time-zero studies (4 mm in diameter) or placed into media for degenerative culture studies (6 mm in diameter).

### 2.4. Time-Zero MeHA Integration and Cartilage Fortification 

To test the integration and mechanical benefits of the tissue-penetrating gel, two separate experiments were performed. In the first experiment (Restoration), cartilage explants underwent three sequential mechanical tests: before any perturbation (“Healthy”), after digestion in collagenase to mimic arthritic changes (“Digested”), and after fortification with MeHA gel (“Reinforced”). For these samples, MeHA gel precursor containing 0.05% *w/v* methacrylated rhodamine was utilized to track diffusion and integration of the gel within the cartilage tissue, as the methacrylated rhodamine is crosslinked to a portion of the MeHA molecules. In the second experiment (Protection), following unperturbed tests, samples were reinforced and tested, followed by digestion and testing. 

#### 2.4.1. Serial Mechanical Testing

For mechanical testing, explants were glued to non-deformable plastic Petri dishes, with the articular surface facing up, and hydrated for at least 60 min in PBS. Explants were mechanically indented with a 1 mm radius spherical probe, applying a creep load of 0.1 N for 10 min [39]. The resulting time, load, and deformation data were analyzed using a Hertzitan Biphasic Theory (HBT) spherical indentation algorithm [40] to quantify biphasic mechanical properties (contact modulus, tensile modulus, and permeability). For all “Digested” groups, explants were digested in 0.1% collagenase type IV at 37 °C for 45 min to model degeneration [41], rinsed in PBS for at least 30 min, and mechanically tested again. For “Reinforced” groups, explants were removed from the PBS, and the superficial cartilage was gently dried with an aspiration line to mimic the suction that would be performed clinically. MeHA (4% *w*/*v*, 15 μL) from one of the three molecular weights (20 kDa, 75 kDa, and 100 kDa) with LAP photo-initiator (0.05% *w*/*v*) was applied to the explant surface, allowed to diffuse for 5 min, and photo-crosslinked for 10 min (400–500 nm, 25 mW/cm^2^). Explants were rinsed 3X with PBS to remove non-crosslinked MeHA, hydrated in PBS for 60 min, and mechanically tested again. For the Restoration experiment, we calculated the recovery percentage (% Recovery) as the amount of change between healthy and digested tissue that is restored following reinforcement (Equation (1)). For the Protection experiment, we calculated a % protection as the final mechanical property (after reinforcement and digestion) divided by the initial.

(1) $$\%Recovery=\frac{\left|Reinforced-Digested\right|}{Healthy-Digested}\times 100$$

#### 2.4.2. Diffusion and Integration with Cartilage Tissue 

For the Restoration samples, the MeHA precursor solution also included methacrylated rhodamine (0.025% *w*/*v*) for visualization of hydrogel interdigitation within cartilage explants. Following the final mechanical test, explants were cut in half axially to yield two hemi-cylinders, fixed in 4% paraformaldehyde (PFA) for 60 min while protected from light, rinsed 3X in PBS, and embedded in optimal cutting temperature (OCT) compound. Frozen samples were sectioned (20 µm section thickness; −20 °C), rinsed in PBS to remove OCT, mounted with ProLong Gold with DAPI, and coverslipped. Sections were imaged via confocal microscopy, and the mean intensity in the red channel (corresponding to rhodamine) in the top 50 μm was quantified. Three images were obtained and quantified from each of the six explants for all three MW MeHA groups.

### 2.5. Living Explant Degenerative Culture 

Following sterile harvest, cartilage explants were cultured in chemically defined media (CDM; Dulbecco’s Modified Eagle’s Medium [DMEM], 50 µg/mL ascorbate-2-phosphate, 40 µg/mL l-proline, 1 mM sodium pyruvate, 0.1% *v/v* insulin-transferrin-sodium selenite premix, and 1% *v/v* penicillin-streptomycin-fungizone [PSF]) for two days as an in vitro normalization period. Explants were obtained from three donors and divided equally amongst five groups (*n* = 3–5 per group per donor). Furthermore, explants obtained from medial, central, and lateral locations on the trochlea were allocated to each group, reducing location-dependent influence on observed differences between groups. All explants were cultured in a confined fashion, within 6 mm diameter agarose wells in 24-well plates, to emphasize media exposure to the articular surface. The first group was cultured in CDM for the duration of the study, serving as a control group (Ctl). The remaining four groups were cultured in CDM supplemented with IL-1β (10 ng/mL; human recombinant; Peprotech), and at the initiation of the study, received either no gel (IL-1β) or one of the three MW MeHA gel treatments (20 kDa, 75 kDa, 100 kDa). For gel treatment groups, dry MeHA was sterilized under ultraviolet (UV) light for 30 min and reconstituted at 4% *w/v* with 0.05% *w/v* LAP in sterile PBS. Explants were removed from the media, the articular surface was dried with suction, and MeHA solutions (15 μL) were applied to the articular surface. After 5 min to allow for MeHA diffusion, the gel was crosslinked under blue light for 10 min. Explants were cultured for 2 weeks, with media replacement every two to three days. Media was collected at each feeding time point to quantify sulfated glycosaminoglycan (s-GAG) release. After the 2 week culture, explants were subjected to Hertzian creep indentation testing (as described above), histology, and immunofluorescence staining. 

#### 2.5.1. DMMB Assay

s-GAG is a key cartilage matrix component that is released into the surrounding media during degenerative processes. To determine s-GAG release from explants into the media, a dimethylmethylene blue (DMMB) assay was performed on media collected at days 3, 5, 7, 10, 12, and 14 of the culture. The media was plated in triplicate in a 96-well assay plate (5 μL), followed by the addition of 200 μL of DMMB solution (16 µg/mL DMMB, 0.5% *v/v* ethanol, 2 mg/mL sodium formate, 0.2% *v/v* formic acid in deionized water). Absorbance values at 525 nm were quantified relative to a standard curve of chondroitin sulfate (Sigma). s-GAG release was normalized to the final volume of tissue, and both temporal and cumulative release were calculated. 

#### 2.5.2. Mechanical Testing 

Compressive creep experiments were performed with a spherical indenter, similar to time-zero testing. However, for the degenerative explant cultures, we utilized 6 mm diameter explants and a 2 mm-radius probe, and applied a load of 0.25 N for 15 min. Time, load, and deformation were fit to a Hertzian Biphasic Theory (HBT) equation with an assumption of strain-independent permeability to obtain contact modulus (Ey-), tensile modulus (Ey+), permeability (k), and coefficient of determination (R2). Fits with an R2 below 0.95 were omitted.

#### 2.5.3. Histology and Immunofluorescence 

Following mechanical testing, explants (*n* = 6–9 per group) were fixed in 4% PFA for 24 h and cut axially in half. The first half went through serial dehydration and paraffin embedding, and the second half was embedded in OCT. Paraffin samples were sectioned (7 µm thick), deparaffinized and rehydrated, stained with Fast Green (0.05% *w*/*v*, 5 min), rinsed in acetic acid (1% *v*/*v*, 20 s), stained with Safranin O (0.2% *w*/*v*, 10 min), rinsed with tap water, dehydrated, mounted with Permount, and coverslipped. Low-resolution (2×) images were taken to visualize entire explant cross sections, and high-resolution (20×) of the articular surface were obtained. To quantify s-GAG retention (Safranin O-staining) at the articular surface, high-resolution images were split into red–green–blue channels. Interestingly, the blue channel allowed for the best delineation of loss of Safranin O staining; the blue channel (intensities 0–155) was inverted and the average of this value in the top 50 μm was recorded as a marker of Safranin O retention.

The second half of the explants were sectioned on a cryostat (20 μm thickness) and rinsed in PBS to remove excess OCT. Sections were blocked with 1% *w/v* bovine serum albumin (BSA) in PBS for 30 min, followed by the application of a primary antibody for NITEGE (aggrecan degradation neoepitope; MD Bioproducts, Zurich Switzerland; 1:200) for 60 min. Following 3X rinses in PBS, a solution containing secondary antibody (goat anti-rabbit; 1:200) and CellMask Deep Red (1:1000) was applied for 60 min. After another round of PBS rinses, sections were mounted using ProLong Gold with DAPI and coverslipped. NITEGE intensity in the top 100 μm was quantified.

### 2.6. Statistical Analysis and Experimental Rigor 

To ensure experimental rigor, all studies were performed with explants from at least three donors. Donor was not a statistically significant factor in two-way analysis of variance (ANOVA) testing, allowing us to pool samples for subsequent analyses. Data were first cleared of outliers with a ROUT test, followed by normality testing using a D’Agostino-Pearson omnibus normality test. Parametric and normal datasets were analyzed using a one-way ANOVA with post hoc Tukey’s multiple comparison test. Nonparametric and/or non-normal datasets were analyzed using the Kruskal–Wallis test with post hoc Dunn’s multiple comparison test. All data were presented as dot plots for transparency, and statistical significance was placed into four categories: *p* < 0.05 (*), *p* < 0.01 (**), *p* < 0.001 (***), and *p* < 0.0001 (****). All statistical analyses were performed using GraphPad Prism 9.0.3.

## 3. Results

### 3.1. Higher MW MeHA Forms Stiffer Hydrogel

Methacrylation was verified in all three MeHA, with a degree of modification ranging from 30–55% (Figure 1B). Since concentration and MW appear to have a greater influence on gelation properties than the degree of modification, subsequent experiments utilized these formulations. Hydrogel mechanical testing further demonstrated that increasing concentration and MW increased gel mechanics (Figure 1C). The 100 kDa-6% gels did not fully follow this trend, as issues with solubility and viscosity may have prevented solution homogeneity prior to cross-linking. Thus, experiments were performed using 4% *w/v* formulations of each gel. 

### 3.2. Lower MW MeHA Integrates Better Than Higher MW MeHA

**Figure 2:**
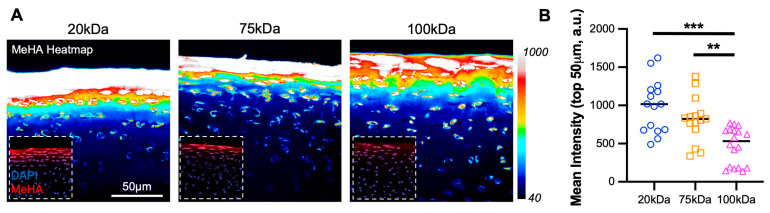
MeHA diffusion into cartilage superficial zone. (**A**) Intensity heatmap (40-1000) of MeHA hydrogel (20, 75, and 100 kDa) within cartilage explants. The inset shows MeHA (red) and DAPI (blue). Scale bar = 50 µm. (**B**) The mean intensity, in arbitrary units (a.u.) of MeHA gel within cartilage in the top 50 µm. *n* = 18 per group. ** and *** represent *p* < 0.01 and 0.001, respectively.

All three MW MeHA’s diffused into and integrated within cartilage tissue, with the 20 kDa HA showing the greatest intensity (Figure 2A). The quantification of intensity in the top 50 µm demonstrated a significant trend of greater material integration with lower molecular weight (Figure 2B), with the 20 kDa MeHA (985.2 ± 346.7 arbitrary units (a.u.)) showing greater than twice the intensity than the 100 kDa MeHA (473.2 ± 241.3 a.u.).

### 3.3. Medium MW MeHA Provides Greatest Restoration of Mechanical Properties 

**Figure 3:**
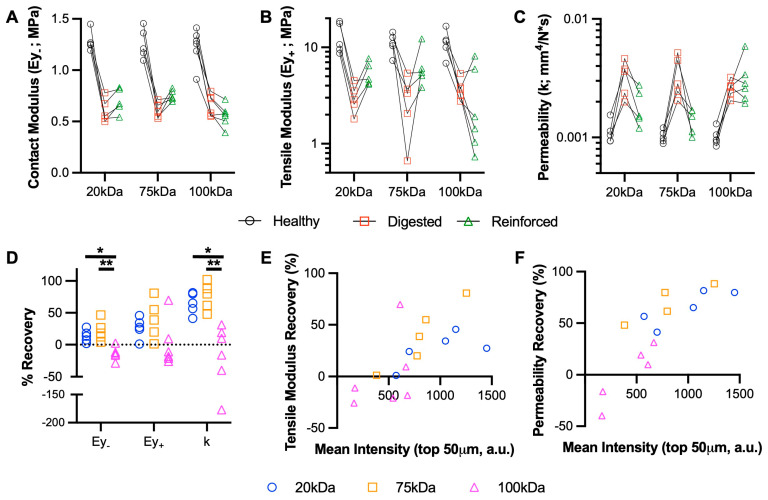
Biomechanical restoration of cartilage using MeHA hydrogels. (**A**) Contact modulus (Ey_-_), (**B**) tensile modulus (Ey_+_), and (**C**) permeability (k) of healthy, digested (collagenase), and reinforced (MeHA) cartilage explants. Points connected by lines represent serial mechanical tests. *n* = 9 per group. (**D**) Percent recovery of explant mechanical properties. * and ** represent *p* < 0.05 and 0.01, respectively. *n* = 9 per group. (**E**) Tensile modulus and (**F**) permeability recovery, plotted vs. MeHA intensity in the top 50 µm for 20, 75, and 100 kDa MeHA gels. *n* = 6 per group.

The digestion of cartilage explants with collagenase demonstrated consistent reductions in contact and tensile modulus and increased the permeability in all three groups of explants. Overall, the 20 kDa and 75 kDa MeHA gels provided a partial restoration of these properties, but the 100 kDa MeHA gel provided no benefit (and in some cases, it led to worsening). For contact modulus specifically (Figure 3A), the 20 kDa and 75 kDa gels led to average increases of 95 kPa (13.7% recovery) and 145 kPa (21.6% recovery), respectively, over previously digested values. The recovery of tensile modulus (Figure 3B) and permeability (Figure 3C) were much more apparent, but not for the 100 kDa MeHA gel. The % recoveries are highlighted in Figure 3D; the 20 kDa and 75 kDa MeHA gels led to 64.8% and 75.9% recoveries in permeability, respectively, both significantly greater than the 100 kDa gel (−28.7% recovery). When the % Recovery in tensile modulus and permeability were plotted against the MeHA intensity in the top 50 µm (Figure 3E,F, respectively), a clear positive relationship was observed.

**Figure 4:**
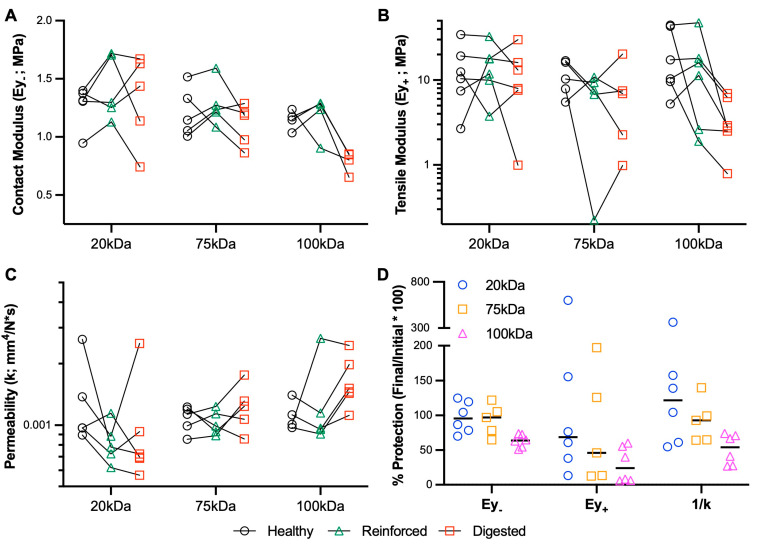
Biomechanical protection of cartilage using MeHA hydrogels. (**A**) Contact modulus (Ey_-_), (**B**) tensile modulus (Ey_+_), and (**C**) permeability (k) of healthy, reinforced (MeHA), and digested (collagenase) cartilage explants. Points connected by lines represent serial mechanical tests. *n* = 4–6 per group. (**D**) Percent protection of explant mechanical properties.

MeHA fortification before digestion was also performed to examine the protective ability of the gel. Overall, this Protection study (Figure 4) resulted in greater variability than the Restoration study. Looking specifically at the % protection in each of the variables (Figure 4D) highlights that the 20 kDa and 75 kDa provide superior protective capacity compared to the 100 kDa gel, especially with regard to contact modulus and permeability. 

### 3.4. Low MW MeHA Provides Greatest Biomechanical Protection during Catabolic Culture

**Figure 5:**
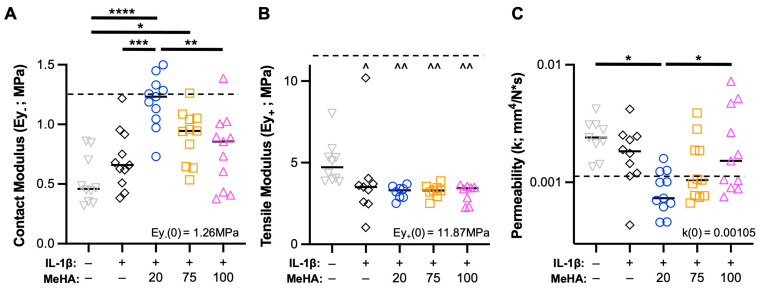
Cartilage explant mechanical properties during IL-1β degenerative culture. (**A**) Contact modulus, (**B**) tensile modulus, and (**C**) permeability of explants after two weeks of culture. *n* = 11 per group from 3 donors. Dashed line represents values from time-zero testing (inset text gives numerical value). *, **, ***, and **** represent *p* < 0.05, 0.01, 0.001, and 0.0001, respectively. ^ and ^^ represent *p* < 0.05 and 0.01, respectively, relative to the Ctl group.

Contrary to prior results [34,42], control explants (Ctl; no IL-1β stimulation) experienced a drastic reduction in contact modulus and tensile modulus, and a sharp increase in permeability, relative to explants at time-zero, and had inferior mechanical properties compared to IL-1β-treated explants. Ctl explants seemed to experience a volumetric expansion, with a significantly greater thickness (4.61 + 0.60 mm) compared to all other groups (average thickness range: 3.39–3.64 mm). The application of MeHA gel at the start of the culture period demonstrated a clear impact of MW (Figure 5), with 20 kDa MeHA gel showing nearly complete protection of the contact modulus (Figure 5A) and permeability (Figure 5C) compared to time-zero samples. In particular, relative to IL-1β treated samples (Ey- = 0.70 MPa, k = 0.00193 mm^4^/N*s), explants treated with 20 kDa MeHA gel (Ey- = 1.19 MPa, k = 0.00089 mm^4^/N*s) showed superior properties. The 100 kDa MeHA gel group showed little-to-no difference in these parameters relative to IL-1β treated samples, with 75 kDa MeHA gel almost exactly between the 20 kDa and 100 kDa groups. Interestingly, all explants treated with IL-1β (including those receiving MeHA gel) had a significantly lower tensile modulus than Ctl samples (Figure 5B).

### 3.5. Low MW MeHA Prevents Superficial Proteoglycan Loss and Matrix Catabolism

**Figure 6:**
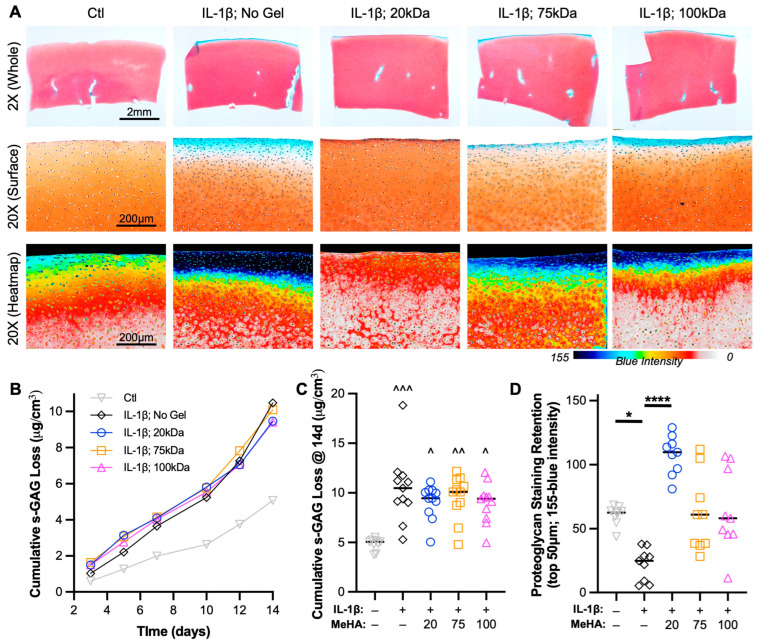
Proteoglycan loss during IL-1β degenerative culture. (**A**) Safranin O-Fast Green stained sections after 2 weeks of culture. (Top) 2×, (Middle) 20×, and (Bottom) 20× blue intensity heatmap images are provided. Scale bars represent 2 mm, 200 μm, and 200 μm. (**B**) Temporal and (**C**) cumulative s-GAG loss quantified via DMMB assay relative to tissue volume. *n* = 11 per group. ^, ^^, and ^^^ represent *p* < 0.05, 0.01, and 0.001, respectively, relative to Ctl. (**D**) Proteoglycan staining retention in the top 50 μm, as measured by the inverse of the blue intensity (155-blue intensity). *n* = 9 per group. * and **** represent *p* < 0.05 and 0.0001, respectively.

Integral to cartilage health is the retention of proteoglycans, which comprise s-GAGs. An analysis of s-GAG release into the media in a temporal (Figure 6B) and cumulative fashion (Figure 6C) showed that all explants treated with IL-1β (including MeHA gel groups) showed more than double the release of s-GAG into the media than Ctl explants. However, given the emphasis on media and MeHA gel application on the articular surface, spatial s-GAG analysis was required. Safranin O staining is a key hallmark of healthy cartilage and chondrogenesis, and loss of staining is typically indicative of a degenerative state. Upon visualization of entire explants (Figure 6A—top row), immediate differences between groups were not observed. However, higher-resolution imaging of the articular surface showed that (1) IL-1β culture led to the depth-dependent loss of proteoglycans, (2) 20 kDa MeHA gel application led to no observable loss of Safranin O staining, and (3) 75 kDa and 100 kDa MeHA gel application showed slight and moderate loss of Safranin O staining, respectively (Figure 6A—middle and bottom rows). This was confirmed by quantification of the inverse of the blue intensity (Figure 6D), which utilizes a lack of visualized Fast Green as a sign of proteoglycan retention. The 20 kDa MeHA gel application consistently facilitated s-GAG retention, whereas the 75 kDa and 100 kDa MeHA gels exhibited a large range of protection from lost Safranin O staining.

**Figure 7:**
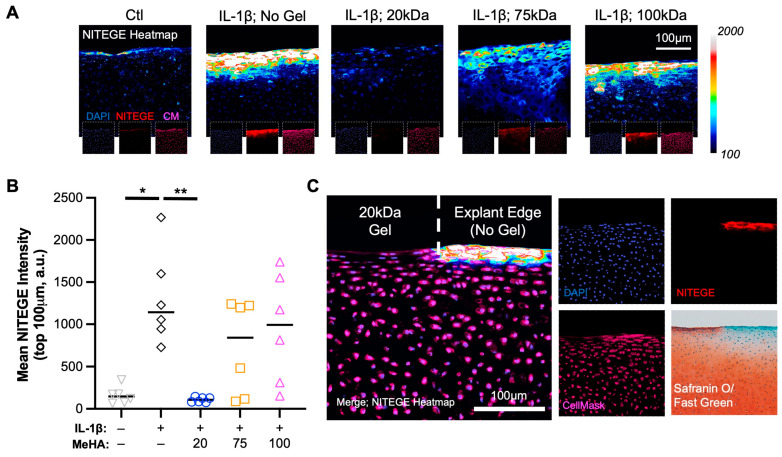
Proteoglycan breakdown (NITEGE) following IL-1β degenerative culture. (**A**) Heatmap of NITEGE staining. Insets are DAPI (blue), NITEGE (red), and CellMask (magenta). Scale bar represents 100 μm. (**B**) Quantification of mean NITEGE pixel intensity in the top 100 μm. *n* = 6 per group. * and ** represent *p* < 0.05 and 0.01, respectively. (**C**) NITEGE heatmap at explant edge, with a clear border between gel-containing and non-gel-containing regions. DAPI, NITEGE, CellMask, and Safranin O/Fast Green staining images. Scale bar represents 100 μm.

To visualize the breakdown of proteoglycans (e.g., aggrecan), explant sections were stained with NITEGE. A clear increase in NITEGE staining was observed from Ctl to IL-1β treated explants; a result completely mitigated using 20 kDa MeHA gel and partially mitigated using 75 kDa and 100 kDa MeHA gels (Figure 7A). The quantification of NITEGE intensity in the top 100 μm confirmed this finding (Figure 7B), with the 20 kDa MeHA gel group showing nearly identical values to the Ctl group. Upon observation of entire explants in the 20 kDa MeHA gel group (Figure 6A—top row, third column), we observed Safranin O staining loss at the corners of the explants that typically receive little-to-no gel due to application restrictions (MeHA precursor application at the edges would cause overflow down the sides of the explant, an issue that would be avoided during clinical use on entire articular surfaces). Visualization of NITEGE staining at the corner (Figure 7C) showed a clear interface between the gel-protected (no NITEGE) and non-protected areas (strong NITEGE), which correspond to positive and negative areas for Safranin O staining, respectively.

## 4. Discussion

Traumatic joint injuries are increasingly common and drastically increase the likelihood of cartilage deterioration. The subtle loss of matrix content and biphasic mechanical properties of cartilage have been confirmed in preclinical PTOA models [17,43], motivating the need for preventative treatments to combat OA progression. To impact both the matrix and cells within, we utilized a tissue-penetrating hydrogel system, which has shown prior success in partially preventing cartilage catabolism. Indeed, tissue-penetrating therapies rely on a host of factors, especially biomaterial molecular weight. Thus, in this study, we aimed to further explore how biomaterial molecular weight impacts the protective effects of a cartilage-infiltrating hyaluronic acid hydrogel, specifically investigating the interplay between biomaterial integration within the tissue and the reinforcement that it provides. Overall, we found that a low MW MeHA gel (20 kDa) provided superior integration (compared to 75 kDa and 100 kDa gels) within the articular cartilage superficial zone, mechanical protection from both collagenase- and IL-1β-mediated degeneration, and biochemical preservation of proteoglycans that are integral to cartilage health.

To maintain a clinically relevant application, short diffusion and crosslinking times (5 min) were utilized. Thus, with limited time for diffusion, the diffusivity of the various MW biomaterials is of great importance. Cartilage porosity and pore size have been reported frequently in the literature, typically in the 6–11 nm range [35,44]. Interestingly, molecules slightly larger than these pores (10–16 nm) have still been shown to diffuse into the tissue [35,45]. Physiological HA and HA viscosupplementation therapies (>2 MDa) likely have calculated hydrodynamic radii well above this pore size (750 kDa HA was calculated to have a 53.9 nm radius [43]), limiting their diffusion into cartilage tissue. However, in this study, we utilized MeHA formulations of 20, 75, and 100 kDa; we performed a regression analysis from values obtained by Kuehl et al. [46], estimating the size of our three MeHA formulations to be 5.78, 13.13, and 15.70 nm, respectively. These theoretical values explain the stark differences between the integration within the cartilage of the 20 kDa gel versus 75 and 100 kDa gels observed in this study. Thus, while the 20 kDa gel on its own may exhibit the lowest mechanical properties of the three formulations, its ability to infiltrate into smaller pores within the matrix may indeed provide more robust fortification as well as increased HA signaling. Certainly, an even lower MW MeHA could have been tested (5 or 10 kDa), but efficient gelation at those MW may be problematic. From our diffusion/integration data, it also appears that all three MeHA formulations showed greater localization around cells, potentially indicating better integration into the pericellular matrix (PCM). Thus, future studies will aim to characterize the relative reinforcements that the MeHA gel provides to the PCM and extracellular matrix (ECM), and how this impacts chondrocyte mechano-transduction.

As mentioned previously, other studies have reinforced cartilage, with chemical crosslinking or biomaterial interdigitation. For example, Makela et al. [29] demonstrated that with an interpenetrating network system (2-methacryloyloxyethyl phosphorylcholine [MPC] and ethylene glycol dimethacrylate [EGDMA] crosslinker), modulus and permeability values (determined via finite element modeling) could be almost entirely restored to native values. Similarly, genipin crosslinking [26] and HA-tyramine [47] systems have provided mechanical fortification of damaged cartilage; however, a few challenges in translating these systems remain, mostly concerning cytotoxicity and application time. Furthermore, these studies did not explore polymer MW or the anti-catabolic effects of these approaches. In our study, we utilized hyaluronic acid to reduce biocompatibility concerns and purposefully maintained low application and crosslinking times. As far as time-zero reinforcement, we demonstrated that the 75 kDa showed the best restoration of mechanical properties following digestion, whereas the 20 kDa gel showed the best protection when applying the gel to healthy tissue (followed by subsequent digestion), potentially because the digestion enabled the larger 75 kDa gel to diffuse and integrate better into the tissue. This may also allow us to tailor specific MeHA applications based on the state of cartilage health (lower MW for healthier tissue and higher MW for more degraded tissue), or perhaps utilize a solution with multiple MW of MeHA to address variable disease states across the joint. Interestingly, the 100 kDa MeHA gel led to the worsening of some mechanical properties. While the excess gel cross-linked above the tissue was removed, this gel may have left residual material on top of the cartilage (due to increased mechanical properties), which led to the apparent softening of the tissue. Finally, our degenerative culture study involved applying the gel at the initiation of the culture, which likely explains why the 20 kDa gel presented the greatest chondroprotective abilities. Future studies will explore the impact of gel application at various points during the culture period. 

Beyond fortification, the other positive aspects of HA cannot be ignored. High MW plays a role in both anti-inflammatory action and improved lubrication [48,49,50], motivating the use of HA injections for early arthritic patients. Interestingly, HA breakdown in the matrix (in vivo and in situ) and the application of low MW HA to cells in vitro can actually lead to a pro-inflammatory response [51,52,53]. Clearly, HA plays a critical and complex role in cartilage homeostasis; the breakdown of HA in the PCM during early PTOA impacts proteoglycan retention, mechano-transduction, as well as anabolic and catabolic pathways [15]. Thus, the implementation of an HA-based tissue-penetrating system, as in this study, can serve to restore HA in the PCM, preventing catabolism, retaining proteoglycans, and maintaining healthy chondrocyte behavior. Future studies will further elucidate the integration of the MeHA into the ECM and PCM, respectively, with electron microscopy, and the effects this has on chondrocyte behavior. Additionally, the comparably persistent nature of the MeHA gel within the cartilage, rather than a one-time injection that may be cleared within hours to days, can also prolong these effects, especially during the critical window of a few weeks following traumatic joint injury. The degree of cartilage protection provided by the 20 kDa MeHA gel in this study mimics some of the other drug delivery and anti-inflammatory approaches from the literature [24,25], giving promise to its future translational approach early in PTOA progression or even immediately after traumatic injury.

The results of this study are promising, but some challenges and limitations were experienced. First, the degeneration of control samples during IL-1β culture, with regard to mechanical properties, was not expected [54]. Upon further inspection, a significant amount of tissue swelling was observed in all three donors, potentially weakening the tissue that appeared to retain its proteoglycan content. Second, we expected the 20 kDa gel to show a deeper diffusion profile than observed; one potential explanation is charge repulsion between the slightly negative HA and significantly negative proteoglycans within the cartilage, which could be combatted in the future with cationic peptide incorporation [23]. A second explanation relates to the penetration of light; the light used to photo-initiate gelation dramatically decreases with cartilage depth [55], limiting its crosslinking. However, given the emphasis on the superficial loss of matrix elements in early PTOA, surface treatment may be adequate in these environments. Third, cytocompatibility (via live/dead staining) was not performed. However, our past studies demonstrated no significant reduction in chondrocyte viability due to the material or photocrosslinking [34,55]. Finally, we acknowledge that the use of juvenile bovine cartilage and IL-1β as a degenerative explant model do not recapitulate the more complex joint environment; our next steps are to evaluate the 20 kDa hydrogel system in an in vivo PTOA model.

## 5. Conclusions

In the present study, we demonstrated that a low MW (20 kDa) MeHA gel effectively penetrates and integrates with the superficial cartilage layer, providing partial restoration of degenerated cartilage and protecting healthy cartilage from subsequent deterioration. The low MW MeHA condition outperformed its higher MW counterparts (75 kDa, 100 kDa) in almost every metric, especially in an IL-1β degenerative explant culture model. We envision that this system could treat PTOA at its start, in that the gel system could be applied during treatment for traumatic injuries (e.g., ACL reconstruction, meniscus repair). Future studies will evaluate this system in vivo and in human OA cartilage, as well as in other musculoskeletal tissues (meniscus and tendon). 

## Data Availability

All raw data are available upon request to the corresponding author.
